# Tissue repair in the central nervous system: the epigenetic programming of regulatory T cells

**DOI:** 10.3389/fimmu.2026.1916267

**Published:** 2026-08-27

**Authors:** Sarah Chenine, Assia Tiane, Paulien Baeten, Janne Verreycken, Stephanie Knippenberg, Oliver Gerlach, Bieke Broux, Daniel LA van den Hove, Tim Vanmierlo

**Affiliations:** 1 Department of Neuroscience, Biomedical Research Institute, Faculty of Medicine and Life Sciences, Hasselt University, Hasselt, Belgium; 2 Department Psychiatry and Neuropsychology, Mental Health and Neuroscience Research Institute, Maastricht University, Maastricht, Netherlands; 3 Academic MS Center Zuyd, Department of Neurology, Zuyderland Medical Center, Sittard-Geleen, Maastricht, Netherlands; 4 Department of Neurology, Maastricht University Medical Center, Maastricht, Netherlands; 5 University MS Center, Campus Diepenbeek, Diepenbeek, Belgium; 6 Department of Immunology and Infection, Biomedical Research Institute, Faculty of Medicine and Life Sciences, Hasselt University, Hasselt, Belgium

**Keywords:** DNA methylation, multiple sclerosis, neuroprotection, regeneration, regulatory T cells

## Abstract

Regulatory T cells are well known for their immunomodulatory role but also their regenerative function is increasingly recognized across multiple tissue types including skeletal muscle, lung and the central nervous system (CNS). In the CNS, Tregs have been shown to promote oligodendrocyte precursor cell differentiation and their interaction with microglia supports a pro-regenerative microenvironment in the brain. The suppressive function of Tregs is highly dependent on the epigenetic signature, specifically DNA methylation, of certain network of Treg-related genes including FOXP3, CTLA4, IKZF2, IKZF4 and TNFRSF18 which collectively function to maintain Treg cell lineage and stability. Whether a similar or the same epigenetic program also influences the regenerative capacity of Tregs remains unknown, and the methylation status of regenerative genes such as AREG, NT3, and osteopontin has never been characterized. Understanding the epigenetic regulation of Tregs in this respect is relevant for demyelinating disease such as multiple sclerosis (MS). MS is characterized by chronic neuroinflammation and neurodegeneration associated with remyelination failure. In MS, Treg suppressive function is compromised and epigenetic dysregulation at key Treg loci has been reported. However, the role of those loci in repair is unknown and the methylation status of regenerative genes in MS Tregs has not been studied. This review examines the evidence for Treg-mediated CNS repair, the epigenetic mechanisms controlling Treg identity, and highlights the epigenetic regulation of Treg regenerative genes as a critical gap in the field with potential implications for remyelination failure in MS.

## 1 Introduction

Regulatory T cells (Tregs) are a specialized subset of CD4+ T cells characterized by expression of forkhead box P3 (FOXP3), interleukin (IL) 2 receptor α-chain (also known as CD25), and specifically in humans, the absence of the IL-7 receptor α chain (also known as CD127) (1, 2). Tregs can be categorized by origin into thymic Tregs (tTregs), which develop in the thymus and possess high affinity for self-antigens, and peripheral Tregs (pTregs), which differentiate from conventional CD4+ T (Tconv) cells in the periphery in the presence of transforming growth factor-β (TGF-β) and IL-2 (3, 4). The primary and most studied function of Tregs is to maintain peripheral immune tolerance and suppress aberrant immune responses (1, 3). Beyond their canonical immunosuppressive role, Tregs have been identified to possess regenerative capabilities across various tissues, including skeletal muscle, lung, and the central nervous system (CNS), through the secretion of anti-inflammatory cytokines and trophic factors that stimulate tissue-resident progenitor cells (5–8). Treg functions, encompassing both immunosuppressive and regenerative roles, are often compromised in autoimmune diseases of the CNS such as multiple sclerosis (MS), Neuromyelitis optica spectrum disorder (NMOSD), and NMDAR antibody (anti-NMDAR) encephalitis (9–13). In these disease conditions, Tregs lose their regulatory capacity, indirectly contributing to the activation of autoreactive T cells and exacerbation of inflammation. In addition, Tregs were shown to interact with glial cells within the CNS, including oligodendrocytes and microglia, to promote remyelination and neuronal repair (7, 14–16).

Epigenetic regulation plays a crucial role in maintaining Treg function and stability, through DNA methylation, histone modifications, and chromatin remodeling, which influence expression of FOXP3 and other proteins key for Treg function (17–19). For example, DNA demethylation at specific sites within the human *FOXP3* locus, particularly at conserved non-coding sequence 2 (CNS2), has been shown to be important for maintaining *FOXP3* expression (18, 19). Demethylation at these regulatory regions enables sustained *FOXP3* expression, thereby reinforcing the suppressive function and lineage stability of Tregs. Several other genes integral to Treg differentiation, function, and stability, such as those encoding cytokines and transcription factors e.g. *CTLA4* and *IKZF2*, are also epigenetically regulated (20–24). The associated epigenetic regulatory network helps to sustain the Treg lineage and enables functional plasticity, which is essential for Tregs’ dual role in immunosuppression and tissue repair. Impairments in epigenetic regulation could disrupt Treg stability and function, potentially exacerbating autoimmune pathology by diminishing their regenerative and regulatory capacities.

This review assesses the emerging literature on Treg regenerative roles in the CNS, including their interactions with glial cells. Additionally, how DNA methylation shapes Treg stability and function is also examined, proposing that epigenetic mechanisms may also govern this regenerative function of Tregs, in addition to their suppressive function. Since MS represents the most prevalent autoimmune disease of the CNS, characterized by both chronic neuroinflammation and remyelination failure, it serves as the central disease model through which the epigenetic regulation of Treg function and regenerative capacity will be explored in this review.

## 2 Regenerative function of Tregs in the CNS

Tissue-resident Tregs have been found to possess regenerative functions and promote repair in tissues such as skeletal muscle, cardiac muscle, lung, skin, and the CNS (7, 25–32). Tregs infiltrate damaged tissues where they not only help resolve inflammation but also actively promote regeneration. They achieve this by secreting specific trophic and mitogenic factors such as amphiregulin (AREG) in muscle and lung tissue and neutrotrophin-3 (NT3) or cellular communication network factor 3 (CCN3) in the CNS (7, 8). These secreted factors directly stimulate the proliferation and differentiation of tissue-resident progenitor cells.

In the CNS, Dombrowski et al. (2017) were the first to elucidate that Tregs are important for remyelination, independent of their immunomodulatory function (7). Remyelination is a dynamic repair process that involves the formation of a new myelin sheath around a previously demyelinated axon. Oligodendrocyte precursor cells (OPCs), a population of multipotent CNS precursor cells, play a central role in axonal repair through their capacity to proliferate, migrate to sites of injury, differentiate, and ultimately mature into myelinating oligodendrocytes that ensheathe axons. Remyelination failure is a hallmark of progressive MS, where chronic demyelination leads to irreversible axonal damage and neurological decline, making the identification of factors that promote OPC differentiation of significant therapeutic interest. In an *in vivo* Foxp3-DTR transgenic mouse model and in *in vitro* brain slice cultures, it was observed that the presence of Tregs results in an increase in differentiated oligodendrocytes expressing myelin basic protein (MBP) (7). Furthermore, it was found that oligodendrocytes cultured with Treg-conditioned medium exhibited a significant increase in differentiated oligodendrocytes in comparison to control conditions. This suggests that Treg-secreted factors influence OPC differentiation and highlights the importance of the regenerative capacity of Tregs in the CNS. The same research group investigated the influence of ageing in the repair capacity of Tregs (33). Tregs were expanded in aged mice *in vivo*, in a lysolecithin-induced demyelination model, by intraperitoneal (i.p.) administration of IL-2/anti-IL-2 complex which resulted in increased frequency of Tregs within the demyelinated regions. Despite increases in Treg frequency at the site of demyelination, aged Tregs failed to promote OPC differentiation and remyelination indicating an intrinsic failure of regenerative capacity in those Tregs. On the other hand, expansion of Tregs from young mice significantly increased the number of differentiated oligodendrocytes *in vivo*, enhanced axonal myelination and OPC differentiation *in vitro* while Tregs of aged mice failed to influence OPC differentiation or remyelination (33). This model captures an intrinsic, age-related decline in Treg regenerative capacity, which may contribute to the remyelination failure observed in progressive MS. However, this aged-Treg profile alone is unlikely to account for the full extent of regenerative failure in progressive disease, where compartmentalized, CNS-intrinsic inflammation, may further impair Treg-mediated repair beyond what age-related decline predicts. Rather than treating Treg aging as equivalent to progressive MS pathology, we propose it as a contributing mechanism operating within a more complex inflammatory environment.

**Figure 1:**
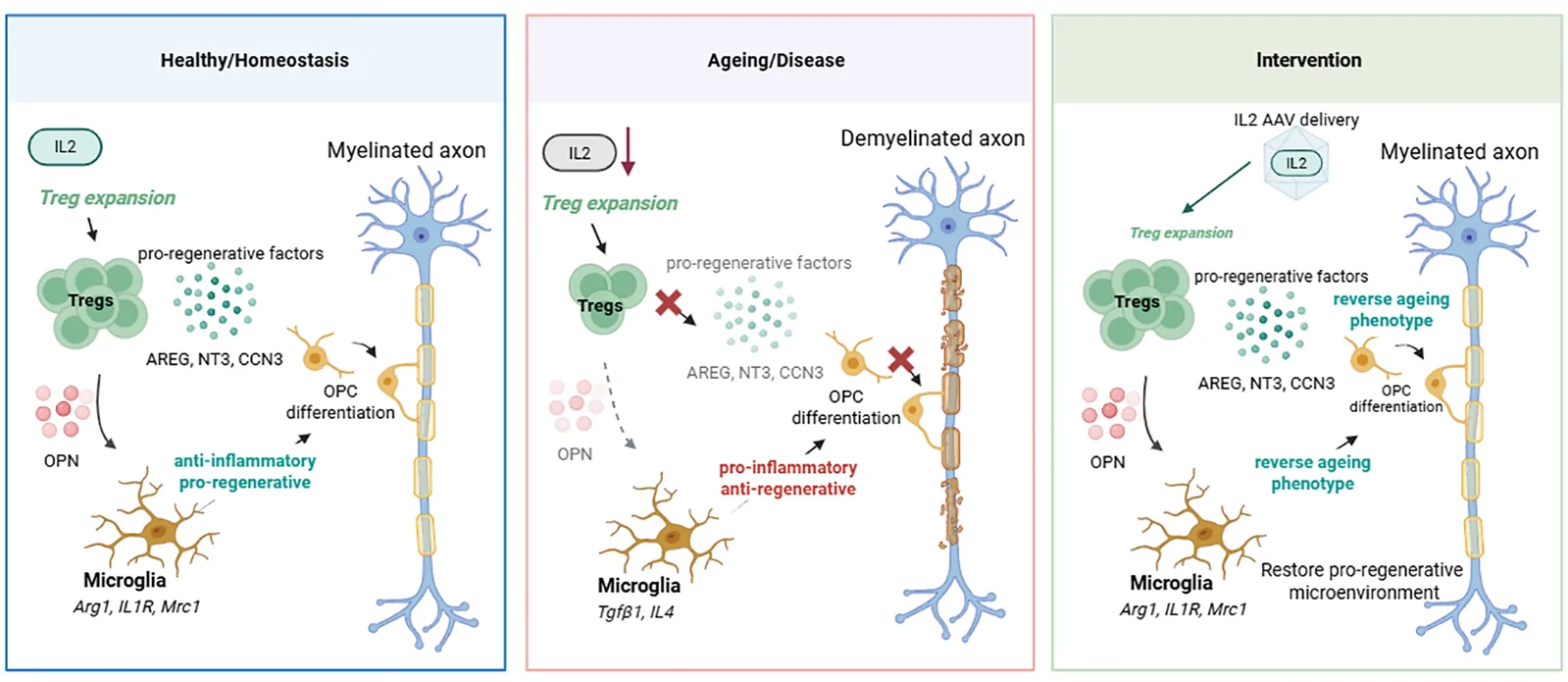
Regulatory T cell-mediated CNS repair under homeostatic conditions and during ageing/disease progression. Under homeostatic conditions, Tregs secrete pro-regenerative factors (AREG, NT3, CCN3) that promote OPC differentiation and myelination. Treg-derived osteopontin (OPN) polarizes microglia towards an anti-inflammatory, pro-regenerative phenotype supporting repair. During ageing and disease progression, Treg regenerative capacity is impaired, and microglia adopt a pro-inflammatory phenotype, contributing to remyelination failure. CNS-targeted IL-2 delivery via an AAV vector restores Treg expansion and reverses age-related transcriptional changes in glial cells, promoting a pro-regenerative microenvironment. Created in BioRender. Chenine, S. (2026) https://BioRender.com/afy1cbl.

There appears to be substantial crosstalk between Tregs and glial cells in repair mechanisms (**Figure 1**). For instance, the presence of Tregs induces microglial polarization towards an anti-inflammatory phenotype. Murine microglia co-cultured with Tregs exhibited increased expression of anti-inflammatory genes, including *Arg1*, *Flg12*, *Mrc1*, and *Il1r* (14). Additionally, Treg cell-conditioned microglia promoted OPC differentiation into mature oligodendrocytes. This pro-differentiating effect is thought to be mediated by the secreted factor osteopontin, which acts through integrin receptors on microglia to enhance their regenerative properties. Interestingly, brain-specific delivery of IL-2 also resulted in upregulated expression of osteopontin, suggesting that Treg-microglia crosstalk enhances the reparative microglial phenotype, which in turn promotes OPC differentiation (14, 34). This Treg-microglia crosstalk is not limited to demyelinating contexts. In spinal cord injury models, Tregs similarly direct microglial function via the osteopontin-CD74 axis to enhance synaptic debris clearance. Additionally, through *Abcg1* upregulation, Tregs regulate microglial cholesterol and lipid metabolism, which is a mechanism critical for processing myelin debris (15). Reciprocally, microglial major histocompatibility complex II (MHC II) expression is required to sustain local Treg numbers and function, collectively supporting tissue preservation and motor recovery (15, 35).

The reparative role of Treg-derived osteopontin described above has been established primarily in models of ischemic injury, such as stroke, rather than in autoimmune demyelination (14, 36). In human MS, osteopontin is instead predominantly characterized as a pro-inflammatory mediator. Elevated cerebrospinal fluid and serum osteopontin levels have been associated with relapses, active lesions, and long-term brain atrophy (37–39). Mechanistically, osteopontin promotes Th1 and Th17 differentiation, suppresses IL-10, and enhances the survival of autoreactive T cells, effects linked to thrombin-cleaved osteopontin fragments that engage the α4β1 integrin, the same integrin targeted by natalizumab (40–42). Whether Tregs themselves contribute to this osteopontin pool in human MS lesions remains unclear. We suggest that osteopontin’s function may depend on the inflammatory context in which it is produced. In chronic, thrombin-rich autoimmune lesions, osteopontin appears to favor pathogenic signaling, whereas in more acute or resolving injury settings, Treg- and microglia-derived osteopontin may favor tissue repair. Further research is needed to determine whether Treg-derived osteopontin contributes to CNS repair in MS specifically, as it appears to in stroke.

Targeting this Treg-glia axis by modulating CNS IL-2 availability can be a promising treatment strategy (34, 43). For brain-resident Tregs, IL-2 is always a limiting factor, limiting their expansion capacity. Delivery of IL-2 via astrocytes using an adeno-associated viral (AAV) vector increased brain IL-2 levels, followed by expansion and increased frequency of the brain-resident Treg population (34). This approach was tested *in vivo* in models of traumatic brain injury, stroke, and multiple sclerosis (34). Neuroprotection was observed in all models after IL-2 induction in the brain, leaving the peripheral immune system unaffected. Additionally, a similar study established that brain IL-2 delivery preserves cognitive function in aging mice (43). Furthermore, co-culture of microglia from aged mice with Tregs supplemented with IL-2 *in vitro* resulted in decreased apoptosis and higher survival rates of microglia. Single-cell RNA sequencing data revealed that treatment with brain-specific IL-2 induced extensive age-related transcriptional changes in glial cells (astrocytes, oligodendrocytes, and microglia), with the ability to reverse almost all aging-related pathways across glial cell types (43). In relation to MS, CNS-targeted IL-2 delivery could boost Treg activity locally without systemic side effects associated with IL-2 therapy. Additionally, given the aging phenotype in progressive MS, IL-2 therapy may serve to reverse age-related transcriptional changes observed in glial cells, restoring a potential pro-regenerative microenvironment for remyelination to occur.

While CNS-targeted delivery strategies remain preclinical, systemic low-dose IL-2 has been tested directly in relapsing-remitting MS in a phase II trial (44). Treatment resulted in activation of Tregs, but expansion was delayed and more modest than observed across other autoimmune diseases (44–46). Notably, this trial assessed inflammatory and Treg activation outcomes only but whether IL-2-activated Tregs also promoted remyelination was not investigated, leaving open whether the regenerative potential suggested by preclinical models translates to MS patients. The trial authors attributed the modest Treg response to the dose and injection schedule used, noting that higher or more frequent dosing has produced stronger effects in other autoimmune diseases (44). This suggests that systemic IL-2 remains a viable strategy in MS, but one that likely requires further dose optimization rather than being an ineffective approach, and reinforces the rationale for CNS-targeted delivery approaches that could achieve local Treg activation without relying on higher systemic doses.

Studies by other groups have shown that, in a human neural stem cell (hNSC) transplantation model in experimental autoimmune encephalomyelitis (EAE), Tregs accumulated in the CNS and activated repair pathways, including remyelination (37). Mice that received the hNSC transplant showed increased Treg numbers and higher myelination than control mice. Importantly, *in vivo* depletion of Tregs abolished the improvement in remyelination despite hNSC transplantation, confirming that this effect was Treg-mediated (47). In a distinct transient middle cerebral artery occlusion stroke model (tMCAO), which reflects ischemic rather than autoimmune injury, administration of activated Tregs (aCD3, aCD28 and IL-2) resulted in increased NSC proliferation compared to inactivated Tregs and control condition. This further highlights the versatile role of Tregs in white matter repair across different injury microenvironments (48).

Specifically in relation to white matter repair, a study by Yuan et al. (2023) has demonstrated that in a tMCAO stroke model, Treg treatment was shown to promote remyelination in late stages (16). MBP staining revealed a progressive loss of white matter, whereas intravenous (i.v.) Treg treatment resulted in a significant decrease in the loss of MBP-positive white matter tissue (16). This suggests that Treg treatment or presence leads to white matter preservation and repair following stroke. A previous study performed by the same group aimed to determine whether Treg-mediated white matter repair was also attributed to its immunomodulatory properties (14). In this study, adoptive transfer of Tregs into Rag1-/- lymphopenic mice resulted in an increase in the number of oligodendrocytes in the ischemic brain, further suggesting that Tregs possess an inherent regenerative function and that lymphocytes are not the mediator of Treg-driven repair mechanisms (14). These findings suggest that, despite the relatively small number of Tregs in the brain, they can influence the brain microenvironment by reprogramming glial cells, and that increasing Treg numbers or activity in the early stages of MS or related neurological conditions may result in long-term improvement in remyelination and disease prognosis.

## 3 Epigenetic regulation of Tregs

Epigenetic mechanisms have been proposed to play a crucial role in determining Treg fate (49). Key epigenetic mechanisms include DNA methylation, histone modifications, and regulatory non-coding RNAs. These mechanisms act in concert to form an intricate network that is essential for regulating cellular processes, cell differentiation, and the normal development of all cell types (50). Among these, DNA methylation is the most extensively studied in the context of Treg biology and represents the most stable and heritable epigenetic mark, making it particularly informative for understanding Treg lineage commitment and functional stability. Specifically, DNA methylation-associated patterns are required for successful Treg differentiation (49). Given that the epigenetic regulation of Treg suppressive function is currently the best-characterized aspect of Treg biology, it provides an important conceptual framework for the following discussion. Rather than serving as the central focus of this review, this section aims to establish the necessary context for understanding the still poorly defined epigenetic mechanisms underlying Treg regenerative function. Although the epigenetic regulation of canonical suppressive markers such as FOXP3 has been extensively studied, it remains unclear whether these same regulatory mechanisms also contribute to the regenerative capacity of Tregs. Accordingly, this section focuses on the role of DNA methylation in shaping Treg identity, stability, and functional properties.

DNA (hydroxy)methylation involves the covalent addition of a methyl group (-CH3) to the C5 position of a cytosine within a cytosine phosphate guanine (CpG) dinucleotide (also referred to as CpG site). DNA methylation is performed by DNA methyltransferases (DNMTs), which add a -CH3 group to cytosine, resulting in 5-methylcytosine (5mC). The DNMT family comprises DNMT1, DNMT3a, and DNMT3b. DNMT1 maintains DNA methylation after replication, while DNMT3a and DNMT3b are responsible for *de novo* DNA methylation. In addition to passive DNA demethylation accompanying cell division, DNA demethylation can also be catalyzed by a family of enzymes called ten-eleven translocation (TET), specifically TET1, TET2, and TET3. TET enzymes catalyze the oxidation of 5mC into 5-hydroxymethylcytosine (5hmC), to 5-formylcytosine (5fC) and 5-carboxycytosine (5caC), and finally to an unmodified cytosine base (50–52). Interestingly, 5-hmC marks have been identified as particularly enriched in the CNS (53–55). Although numerous exceptions exist, generally, in promoter regions, high levels of DNA methylation are thought to result in transcriptional repression, while DNA hydroxymethylation is associated with transcriptional activation (56).

For Tregs to be functional, *FOXP3* must be demethylated in a specific manner. This demethylation occurs at the CNS regions within *FOXP3*, which serve as enhancers and regulators of *FOXP3* expression and Treg stability. The best characterized CNS regions are CNS1, CNS2, and CNS3. More recently, CNS0 was also identified, which is thought to be involved in genome organization and chromatin structure (Kitagawa et al., 2017). In mice, CNS1 has been identified as necessary for pTreg stimulation and does not play a role in tTregs, whereas CNS3 is important for the TCR-Foxp3 signaling. CNS2, first characterized by Zheng et al., is the region that maintains stable *Foxp3* expression and is also known as the Treg-specific demethylated region (TSDR) (57, 58). This region is primarily regulated by DNA demethylation, which is necessary for stable *Foxp3* expression and Treg suppressive function (57). Although the core principle of epigenetic stabilization of FOXP3 expression appears to be conserved across species, the regulatory architecture of the FOXP3 locus is not fully equivalent between mice and humans. The role of the murine Foxp3 CNS2 in maintaining stable Foxp3 expression has been well established, and the human TSDR represents the orthologous element which is evolutionarily conserved between species (57, 59, 60). However, additional cis-regulatory elements and transcription factor networks within the human FOXP3 locus may contribute to species-specific regulation of FOXP3 expression and Treg stability (61). Moreover, human conventional CD4+ T cells can transiently express FOXP3 upon activation, underscoring the importance of TSDR demethylation as an indicator of stable Treg lineage commitment rather than relying on FOXP3 expression alone (60, 62). Thus, while fundamental epigenetic principles governing FOXP3 stability are broadly conserved, the precise regulatory circuits controlling human Treg identity may not be fully recapitulated by murine models.

When demethylated, several transcription factors, including *Ets1, Gata-3*, *Stat5, CREB, Runx, NFAT, c-Rel, AP-1*, and *Foxp3* itself, assemble at the *Foxp3*-binding sites and activate *Foxp3* expression (63). The demethylation level of the TSDR significantly influences Treg function, particularly their suppressive function (57, 64, 65). This is especially evident in murine *in vitro-*generated Tregs (iTregs), which possess a hypermethylated TSDR, leading to loss of phenotype and function and rendering them unstable (66). Cameron et al. (2020) used CRISPR/dCas9 to induce demethylation at the CNS2 region in *in vitro-*generated murine Tregs, resulting in improved stability and suppressive capacity of those Tregs (58, 67). However, Kressler et al. (2021) demonstrated that demethylation of the TSDR may be sufficient to induce *FOXP3* expression in conventional human CD4+ T cells, yet insufficient to trigger a phenotypic switch of CD4+ T cells into functional Tregs (19). This suggests that *Foxp3* expression alone is not a sufficient Treg-specific marker, and that additional transcription factors or epigenetic signatures are also necessary. To further validate this, Ohkura et al. (2012) demonstrated that retroviral overexpression of *Foxp3* in murine CD4+ Tconv cells did not result in the demethylation of the necessary Treg signature genes required for Treg function (68). This suggests that FOXP3 expression alone is not sufficient Treg-specific marker, and that additional transcription factors or epigenetic signatures are also necessary.

**Figure 2:**
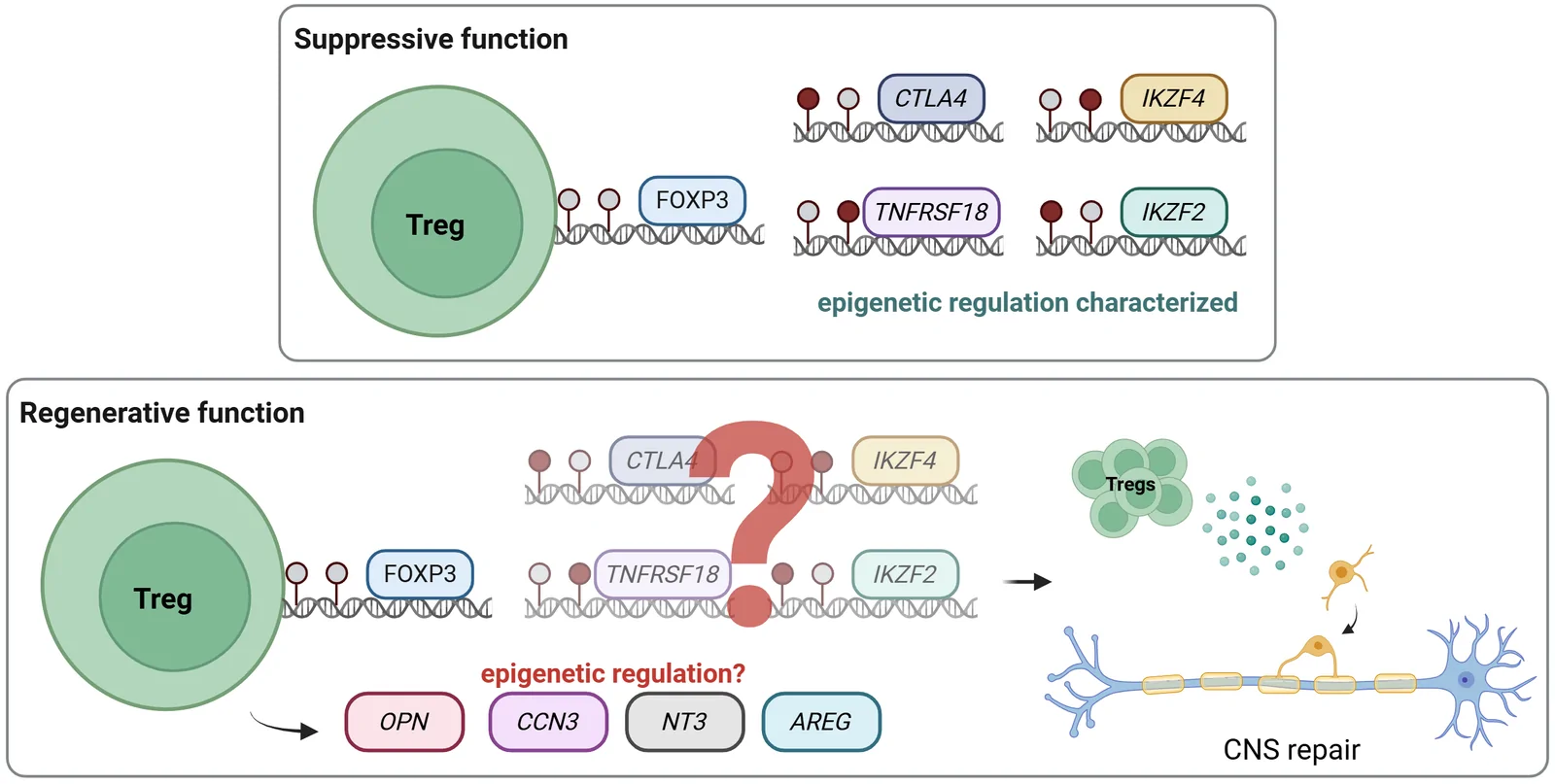
Epigenetic regulation of Treg suppressive and regenerative function. Treg suppressive identity is maintained by differential methylation of signature genes including *FOXP3, CTLA4, TNFRSF18 (GITR)*, *IKZF2 (Helios)*, and *IKZF4 (Eos)*. Whether similar epigenetic mechanisms regulate the regenerative capacity of Tregs remains unknown. Treg-secreted factors implicated in CNS repair such as *OPN, CCN3, NT3*, and *AREG* have not been characterized with respect to their methylation signatures, representing a key gap in the field. Created in BioRender. Chenine, S. (2026) https://BioRender.com/11eoa53.

In fact, a Treg-specific methylation signature that is independent of *Foxp3* expression is essential for Foxp3+ T cells to attain a stable genome-wide Treg expression pattern. This Treg-specific methylation signature includes Treg-signature genes such as *Ctla4, Ikzf2 (Helios), Ikzf4 (Eos), and Tnfrsf18 (GITR*) (17, 18, 69, 70). Expression of these genes has been illustrated at the Treg precursor stage, well before the onset of *Foxp*3 transcriptional activity. It is known that this specific set of genes (*Ctla4, Ikzf2, Ikzf4, and Tnfrsf18)*, including *Foxp3*, is regulated by DNA methylation, forming the Treg epigenetic signature required for stable Treg development and function (69) (**Figure 2**).

For example, Tregs acquire *Ctla4* demethylation during development at a precursor stage through a mechanism that is usually enhanced by IL2 (71). Additionally, *Ctla4* exons, specifically exon 2, exhibit a distinctive methylation pattern in Tregs compared to Tconv cells (17). Furthermore, in patients with myasthenia gravis, the *CTLA4* promoter region showed increased methylation, leading to lower *CTLA4* mRNA levels and decreased Treg-associated cytokines, such as IL-10 and TGF-β (72). Similarly, in the *TNFRSF18* gene, exon 4 has been confirmed to be totally methylated in Tconv cells compared to Tregs (73). It has been observed that this results in lower *GITR* mRNA levels in Tconv compared to Tregs. Furthermore, exon 5 of *Tnfrsf18* is hypomethylated in murine Tregs and can serve as a distinguishing feature between Tregs and other T cell types. For example, only tTregs show this pattern in exon 5 of *Tnfrsf18*, whereas pTregs are hypermethylated at this locus (71). In addition, two members of the Ikaros transcription factor family, namely IKZF2 (Helios) and IKZF4 (Eos), are established Treg signature genes important for the stability and function of Tregs. They display a hypomethylation signature in Tregs, primarily in their gene bodies, rather than in promoter regions. Specifically, in mice, *Ikzf2 (Helios)* has been observed to display extensive demethylation in intron 3a and exon 6, whereas *Ikzf4 (Eos)* shows demethylation in intron 1b in tTregs compared to Tconv cells (68). *Ikzf2, Ikzf4*, and *Tnfrsf18* show expression peaks during the Treg precursor stage before *Foxp3* induction, indicating that their expression is independent of *Foxp3* expression (24). *Ikzf4* is required for *Foxp3* to inhibit its downstream targets, e.g., inflammatory cytokines, to maintain the suppressive phenotype of Tregs (74). It has therefore been shown that downregulation of *Ikzf4* may reprogram tTregs into a T helper-like phenotype, even though *Foxp3* levels remain unchanged, indicating the importance of stable *Eos* regulation and expression for Treg function (74). In addition, *Ikzf2* and *Ikzf4* both regulate the *Il2* promoter through epigenetic mechanisms (20, 22). Knockdown of *Ikzf4* results in demethylation at the *Il2* promoter region upon CD3 and CD28 stimulation (Pan et al., 2009). Similarly, *Ikzf2* binds to the Il2 promoter to maintain its histone deacetylation, resulting in epigenetic silencing of *Il2* (22). *Ikzf2-* or *Ikzf4-*deficient Tregs lose their capacity to suppress immune responses and develop unstable phenotypes during inflammatory responses (20, 22, 75).

This information altogether demonstrates that the DNA methylation signature of Tregs is highly important for their function and stability. The Treg epigenetic program extends well beyond the FOXP3 locus, encompassing a network of signature genes including CTLA4, IKZF2, IKZF4, and TNFRSF18, whose coordinated demethylation is required for stable Treg lineage identity and suppressive capacity. While most research to date has investigated how epigenetic regulation influences suppressive Treg function, the biology underlying Tregs’ regenerative capacity remains unknown. Treg-associated secreted factors such as osteopontin, NT3, AREG, and CCN3 may also be subject to epigenetic regulation in a manner like that of the suppressive genes, yet their methylation signatures have not been characterized in any disease context.

## 4 Implications for multiple sclerosis

MS is a chronic autoimmune demyelinating disease of the CNS, characterized by autoreactive T cell-mediated attack on myelinated axons, progressive neuroinflammation, and the gradual failure of endogenous repair mechanisms (76). Effector T cells, mostly T-helper17 (Th17) cells, have been implicated in the pathogenesis of MS, resulting in critical damage to myelinated axons of the CNS (77, 78).

The primary observed pathology in MS is the presence of focal demyelinated regions induced by autoimmunity (76, 79). In homeostatic conditions and during relapse-remitting MS (RRMS), OPCs retain the ability to differentiate into myelinating oligodendrocytes. However, in progressive MS (pMS), the innate ability of OPCs to differentiate and remyelinate decreases and is eventually lost (80). At the root of MS neuropathology, levels of myelin-reactive CD4+ T helper 1 and 17 cells (Th1 and Th17) increase, while the population of regulatory T cells (Tregs) which modulate immune function decreases, disrupting peripheral self-tolerance mechanisms. In MS, Treg frequencies have been shown to be altered, and their suppressive function is less optimal (12, 13, 81). Since suppressive function is affected by disease, this may also be reflected in their regenerative function, although no research to date has specifically examined this. The observation that aged Tregs lose their regenerative capacity in demyelination models and that Treg depletion impairs OPC differentiation and white matter integrity raises the question of whether a similar intrinsic failure of regenerative capacity occurs in MS Tregs as a form of epigenetically induced premature aging (7, 14, 43).

Epigenetic dysregulation may explain a proportion of Treg dysfunction in MS. In an EAE mouse model of MS, the *Foxp3* promoter was hypermethylated compared with control mice (82). Similarly, in RRMS patients, *FOXP3* mRNA and protein levels were diminished, suggesting that *FOXP3* methylation may influence its expression and, therefore, Treg function in MS patients (83, 84). Additionally, a study conducted in pregnant RRMS patients also identified epigenetic modifications on the promoters and enhancers of the *FOXP3* locus (85). A recent study reported decreased *IKZF2* expression in RRMS patient Tregs compared to controls (86). As discussed previously, IKZF2 is a core component of the Treg-specific epigenetic signature and is essential for lineage stability and suppressive function. Whether this decrease reflects direct epigenetic dysregulation at the IKZF2 locus in MS Tregs remains to be determined.

Treg dysfunction has similarly been implicated in other neurological conditions, including Alzheimer’s disease, anti-NMDAR encephalitis, amyotrophic lateral sclerosis, NMOSD, and stroke, yet the epigenetic basis of this dysfunction remains largely unexplored across all these contexts (9–11, 87–90). Dysfunctional and epigenetically altered Tregs may contribute to disease pathology and progression, emphasizing the need for further research and the exploration of Treg-modulating repair therapies.

## 5 Discussion

Taken together, much of what is known about Treg-mediated CNS repair and the epigenetic regulation of Treg identity comes from murine models of demyelination, ischemic injury, and autoimmunity. These models cannot fully capture the compartmentalized inflammation, chronic antigen exposure, and clinical heterogeneity of MS. This gap is likely part of why Treg-based therapies have so far translated less successfully than preclinical data would suggest, as illustrated by the more modest and delayed Treg expansion observed with systemic low-dose IL-2 in relapsing-remitting MS (44). It also remains unclear whether murine findings on Treg-glia crosstalk, OPC differentiation, and age-related regenerative decline hold in human Tregs within the MS-affected CNS, and as discussed above, some of the regulatory circuitry governing Treg identity itself differs between species. This translational gap remains a key challenge for advancing Treg-based therapies toward clinical application in MS.

This review has examined two converging bodies of evidence: the emerging role of Tregs as active mediators of CNS repair, and the epigenetic mechanisms that govern Treg identity and stability. Based on the evidence reviewed here, we propose that epigenetic dysregulation in MS Tregs may extend beyond genes governing suppressive function to also influence the regenerative gene program, potentially contributing to remyelination failure in demyelinating diseases, specifically in progressive MS.

A key message of this review is the importance of broadening the epigenetic focus beyond FOXP3 as the primary Treg lineage marker. While FOXP3 has long been considered the defining marker of Tregs, the evidence reviewed here demonstrates that other Treg signature genes, including *CTLA4, IKZF2, IKZF4*, and *TNFRSF18*, are equally as important for Treg lineage stability and function, and are independently subject to epigenetic regulation. Importantly, whether the dysregulation of these genes in MS also has consequences for Treg regenerative capacity remains undetermined. Similarly, the methylation status of repair-associated factors such as osteopontin, NT3, AREG, and CCN3 in MS Tregs and healthy donor Tregs has never been characterized.

From a therapeutic perspective, the findings reviewed here highlight several potential treatment options. CNS-targeted IL-2 delivery via AAV-mediated astrocyte expression has demonstrated the ability to expand brain-resident Tregs and promote glial reprogramming in models of MS, stroke, and traumatic brain injury, while leaving the peripheral immune system unaffected. Adoptive transfer of ex vivo expanded Tregs has shown early promise in promoting remyelination and OPC differentiation in preclinical models, though ensuring the regenerative competence of expanded Tregs remains a key challenge. Looking further ahead, CRISPR/dCas9-mediated epigenetic editing at key Treg loci, as demonstrated at the FOXP3 CNS2 region, offers a route to restoring both suppressive stability and potentially regenerative function in MS Tregs (19, 91, 92). This approach remains speculative for regenerative genes specifically, since their methylation status remains uncharacterized in MS, and therefore represents a more distant therapeutic prospect. Overall, understanding how epigenetic modifications influence Treg maintenance, stability, and function offers insights into potential targets for restoring immune balance and possible Treg-mediated repair therapies.
